# Energy-entropy multiscale cell correlation method to predict toluene–water log *P* in the SAMPL9 challenge†

**DOI:** 10.1039/d3cp03076h

**Published:** 2023-09-25

**Authors:** Hafiz Saqib Ali, Richard H Henchman

**Affiliations:** a Chemistry Research Laboratory, Department of Chemistry and the INEOS Oxford Institute for Antimicrobial Research, University of Oxford 12 Mansfield Road Oxford OX1 3TA UK hafiz.ali@chem.ox.ac.uk; b Sydney Medical School, Faculty of Medicine and Health, University of Sydney Sydney Australia rhen7213@uni.sydney.edu.au

## Abstract

The energy-entropy multiscale cell correlation (EE-MCC) method is used to calculate toluene–water log *P* values of 16 drug molecules in the SAMPL9 physical properties challenge. EE-MCC calculates the free energy, energy and entropy from molecular dynamics (MD) simulations of the water and toluene solutions. Specifically, MCC evaluates entropy by partitioning the system into cells of correlated atoms at multiple length scales and further partitioning the local coordinates into energy wells, yielding vibrational and topographical terms from the energy-well sizes and probabilities. The log *P* values calculated by EE-MCC using three 200 ns MD simulations have a mean average error of 0.82 and standard error of the mean of 0.97 *versus* experiment, which is comparable with the best methods entered in SAMPL9. The main contribution to log *P* is from energy. Less polar drugs have more favourable energies of transfer. The entropy of transfer consists of increased solute vibrational and conformational terms in toluene due to weaker interactions, fewer solute positions in the larger-molecule solvent, reduced water vibrational entropy, negligible change in toluene vibrational entropy, and gains in solvent orientational entropy. The solvent entropy contributions here may be slightly underestimated because software limitations and statistical fluctuations meant that only the first shell could be included while averaged over the whole solution. Nonetheless, such issues will be addressed in future software to offer a general method to calculate entropy directly from MD simulation and to provide molecular understanding or guide system design.

## Abstract 2

The energy-entropy multiscale cell correlation (EE-MCC) method is used to calculate toluene–water log *P* values of the 16 drug molecules in the SAMPL9 physical properties challenge.

## Graphical abstract

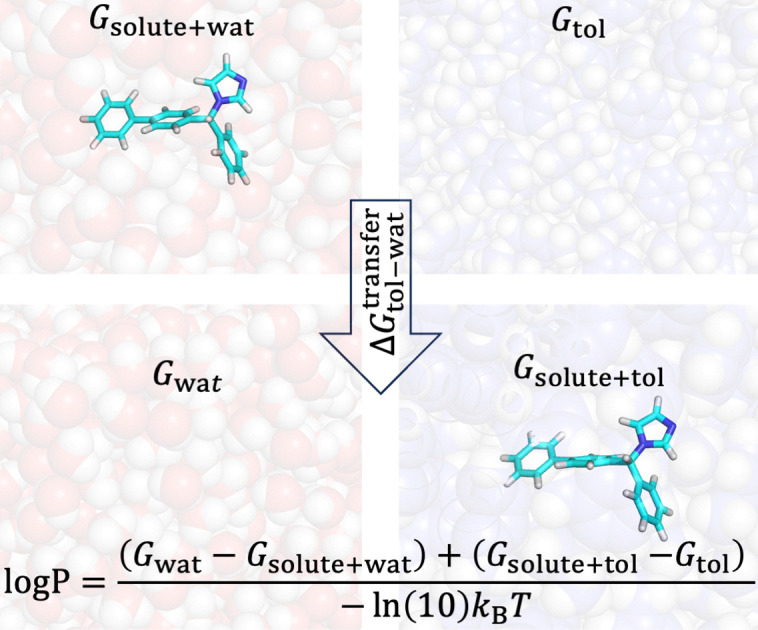

## 1. Introduction

The base-10 logarithm of the partition coefficient *P* of a molecule, log *P*, represents the degree of dissolution of a molecule in one immiscible liquid relative to another. One liquid is typically polar, usually water, and the other non-polar, making log *P* a measure of a molecule's hydrophilicity or hydrophobicity.^1^ This property is highly significant in assessing a molecule's bioavailability, toxicology, and pharmacological suitability, and it is, for example, part of Lipinski's rule of five.^2,3^ The organic phase mimics the cell membrane that drugs would need to cross from one aqueous compartment to another. Most commonly, the partition coefficient is measured from water to octanol.^4,5^ Other non-polar solvents include chloroform, alkanes such as *n*-dodecane or *n*-hexadecane, 1,2-dichloroethane, dibutyl ether, cyclohexane, toluene, and propylene glycol dipelargonate.^6–12^ Low solubility in solvents such as alkanes limits the applicability of log *P*,^13–15^ although drugs that are flexible may be able to adopt particular conformations that differentially optimise their surface interactions with a particular solvent^11^ or stabilise intramolecular interactions, information that may prove useful in adjusting log *P* to desired therapeutic ranges.^16^

Experimentally, log *P* is directly measured from the ratio of the concentrations of the molecule in the two liquids. Computational methods to predict log *P* are also widely used, offering advantages in speed or molecular insight, but not always with clear-cut accuracy and reliability. Knowledge-based and machine-learning methods are fast and widely used after training on databases of known log *P* values for specific solvents.^1,13,14^ Electronic structure methods calculate the solvation free energy by treating the solute at the electronic structure level, the solvent as a dielectric continuum, and the interface between them with atomic surface tension parameters.^17–19^ These include the quantum mechanical self-consistent reaction field (QM-SCRF)^17–19^ and the conductor-like screening models (COSMO).^20,21^ Alchemical methods are a widely used route to log *P*, calculating it from the solvation free energy in each liquid using a series of molecular dynamics (MD) simulations that gradually decouple the solute from the solvent.^22,23^ They typically use all-atom solute and solvent modelled with force-field parameters. Methods that have been used to calculate log *P* directly from a simulation of each solution without intermediate states include linear interaction energy (LIE),^24^ the three-dimensional reference interaction site model (3D-RISM),^25^ grid inhomogeneous solvation theory (GIST),^26^ and energy-entropy multiscale cell correlation (EE-MCC).^27^

**Fig. 1:**
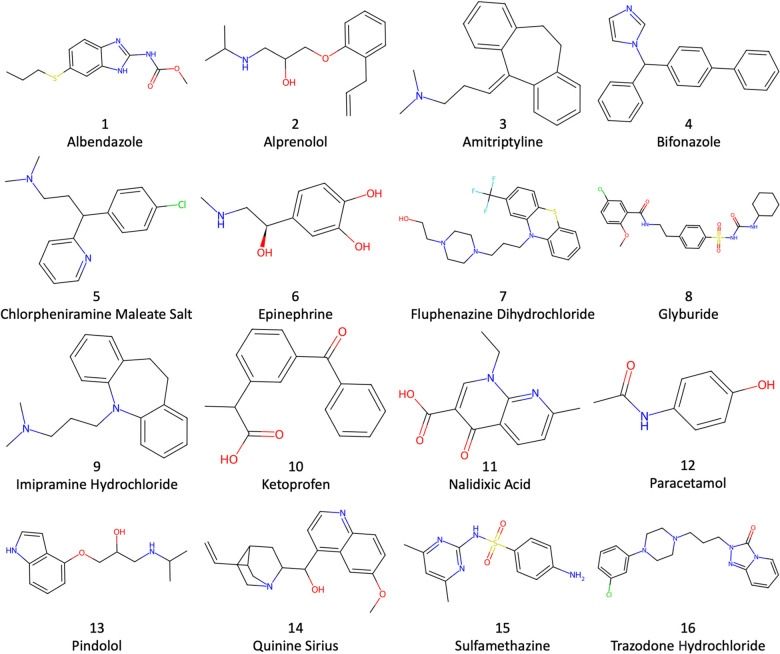
Structures of the 16 SAMPL9 log *P* drug molecules.

To help assess the accuracy and capabilities of different computational methods, the SAMPL (statistical assessment of the modelling of proteins and ligands) log *P* blind challenges have helped demonstrate the performance of computational methods.^28^ Previously they involved water with octanol or cyclohexane solvent. Now in the ninth running, the SAMPL9 log *P* challenge involves the less commonly used toluene–water log *P*^6–12^ for the drug molecules depicted in Fig. 1.

In this work we apply and further test the EE-MCC method used previously to calculate octanol–water log *P* values,^29–31^ this time to the toluene–water log *P* values in the SAMPL9 log *P* Challenge. In EE-MCC the energy is taken from the MD simulations and the entropy is calculated for both solute and solvents over all degrees of freedom at multiple length scales. MCC has been developed for liquids,^29,30,32^ solutions,^33–36^ chemical reactions,^37^ host–guest systems,^38^ and proteins^31,39–41^ and offers the advantage of providing a comprehensive breakdown of entropy across all atomic degrees of freedom.

## 2. Methods

### 2.1 EE-MCC log *P* Calculation

The log *P* value for a solute partitioning from water to toluene relates to the transfer Gibbs free energy Δ*G*^transfer^_tol–wat_ by

1 log *P* = −Δ*G*^transfer^_tol–wat_/(ln(10)*k*_B_*T*)

where *k*_B_ is Boltzmann's constant and *T* is temperature. This equals the Gibbs free energy of the solute in toluene and pure water minus that of pure toluene and the solute in water

2 Δ*G*^transfer^_tol–wat_ = (*G*_sol+tol_ + *G*_wat_) − (*G*_tol_ + *G*_sol+wat_)

where sol + tol and sol + wat denote the solute in the respective solvents toluene or water, and wat and tol denote the respective pure liquids. The solutions are assumed to be dilute and are defined to have the same concentration, such that the transfer energy does not depend on the solute concentration. We ignore the small amount of solvent mixing that takes place for experiment. In the EE method, *G* of each system is calculated using the standard thermodynamic expression *G = H*–*TS*, where *H* is enthalpy, *S* is entropy and *T* is temperature. *H* is directly obtained from a MD simulation as the average potential energy plus the average kinetic energy of the system, and the pressure-volume term is ignored because it is small at ambient pressures and even then cancels in the difference. *S* is calculated from the same MD simulation using the MCC method, which is described in the next section. Four simulations are required for a single log *P* calculation, but the pure solvent simulations are identical, and so effectively only two simulations per solute are required.

### 2.2 Multiscale cell correlation

Entropy is calculated from an MD simulation using MCC in a multiscale fashion in terms of cells of correlated units. Local coordinates are defined for different structural levels of each molecule. Each coordinate is partitioned into discrete energy wells. This gives rise to a vibrational term from the average energy well and a topographical term from the probabilities of each energy well. The total entropy is calculated as the sum of the vibrational and topographical terms for each molecule type, multiscale level and coordinate using3
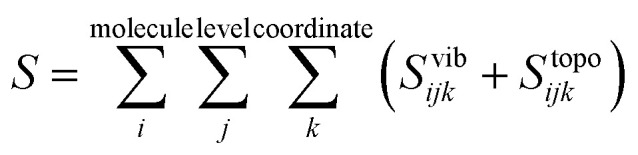
Applied to the case of a solute in solvent, eqn (3) becomes4
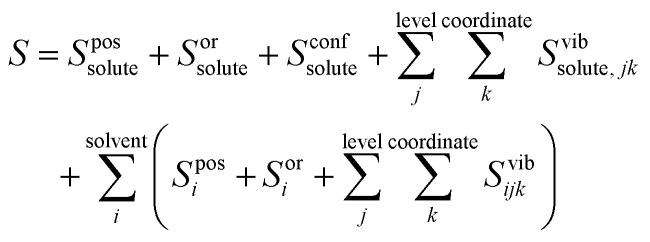
where *S*^pos^_*i*_, *S*^or^_*i*_ and *S*^conf^_*i*_ are positional, orientational and conformational topographical terms, and *S*^vib^_*ijk*_ are the vibrational terms. Next we explain what these terms are and how to calculate them.

#### Molecule decomposition

MCC derives effective potentials for each molecule in the mean field of its neighbours, justified by the weak and diffuse nature of the multimolecular correlations. This is made possible by calculating entropy from molecular forces which may be partitioned in a mean-field manner, as discussed later. This allows entropy to be conveniently and intuitively decomposed according to each molecule. The two types of molecule in a solution are the solute and solvent. There is typically only a single solute which is the drug molecule. Solvent entropies for solutions and pure liquids are calculated by averaging over all solvent molecules but only the contribution from the number of molecules in the first solvation shell is included because the entropies over all solvent molecules are not well-converged and have excessive noise. Solvation shells for each solute are determined using the relative angular distance (RAD) algorithm with the position of each molecule being defined by its center-of-mass.^42,43^

#### Level decomposition

For each molecule, MCC uses a hierarchical coordinate system, treating each molecule as a separate rigid body and decomposing it into collections of smaller rigid bodies. This enables an efficient and scalable calculation of entropy because it separates out larger-scale motion from smaller-scale motion that is difficult to include in the same single coordinate system at one length scale. Moreover, it more naturally captures multiscale motion and non-linear motion such as rotation. The two levels used here are united-atom (UA) and monomer (M). A UA comprises each non-hydrogen atom and its bonded hydrogens. A monomer is defined as an assembly of covalently bonded UAs. Water has only the UA level while toluene and the drugs, comprising multiple UAs, have both levels. Along with previous work,^42,43^ we do not use the “molecule” level, which is inconsistent for molecules having different numbers of levels. The more detailed “atom” level is not considered because this involves high-frequency motion of light hydrogen atoms, which are strongly quantised to essentially single energy levels at room temperature.

#### Coordinate decomposition

For a given molecule and level, entropy is decomposed along the relevant coordinates. At the M level the coordinates are three translations and three rotations, which are defined using the principal axes with the origin at the centre of mass of the molecule. Being orthogonal coordinates as eigenvalues of a covariance matrix, in this case a force covariance matrix, their entropy may be evaluated separately. At the UA level, translation involves the collective motion of covalently bonded UAs in the M coordinate frame, motion that can also be regarded as internal motion at the M level. A non-linear molecule with *N* UAs has 3*N*-6 coordinates of collective motions. Again, entropy can be evaluated separately along each coordinate because they are eigenvectors of a covariance matrix. Concerning rotational motion, a UA with two or three hydrogens is non-linear and has three degrees of freedom, with one hydrogen it is linear and has two rotational degrees of freedom, and with no hydrogens it is a point and has no rotational degrees of freedom. The coordinate system for UA rotation has the origin at its heavy atom and the axes are determined according to the covalent bonds to neighbouring atoms as defined elsewhere.^29^

#### Vibrational entropy

The vibrational entropy relates to the average size of the energy wells along a given coordinate *k*, level *j* and molecule *i*. It is calculated for each vibration *ijk* in the harmonic approximation using the equation for a quantum harmonic oscillator5
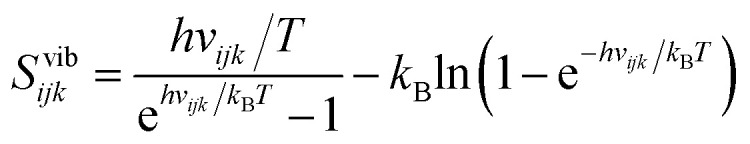
where *k*_B_ is Boltzmann's constant, *h* is Planck's constant, and *v*_*ijk*_ is the vibrational frequency, which is derived from the eigenvalue *λ*_*ijk*_ of a covariance matrix using6
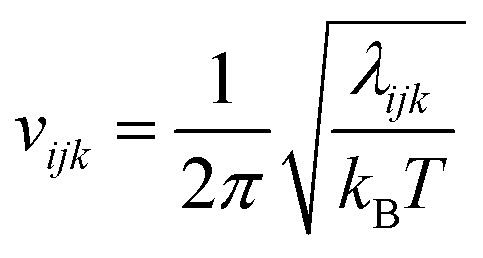
For translation the matrix is the mass-weighted force covariance matrix, with elements given by 
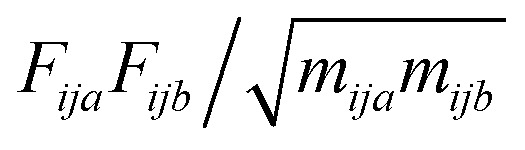
, where *F* is force, *m* is mass, and *a* and *b* are indices over coordinates. At the M level the matrix is 3 × 3 using the principal axes of M. At the UA level it is 3*N* × 3*N* for the *N* UAs in the principal axes of M, and requires the removal of the six lowest-frequency vibrations to avoid double-counting translational and rotational entropy already determined at the larger M level. We use the term “transvibrational” to denote translational vibrations. For rotation the matrix is the moment-of-inertia-weighted torque covariance matrix with elements given by 
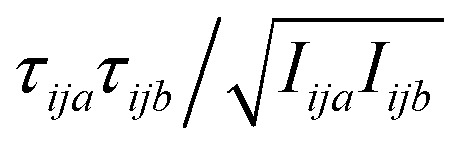
, where *τ* is torque and *I* is moment of inertia. At the M level the matrix is 3 × 3 and involves rotation about the principal axes of M. At the UA level it is a matrix with dimension calculated by summing over the number of rotations for each UA: 3 for non-linear UAs, 2 for linear UAs and zero for point UAs. We use the term “rovibrational” to denote rotational vibrations. The forces and torques in both M matrices and in the UA rotational matrix are halved in the mean-field approximation^29,30,37,38,44^ because the interacting atoms are negligibly correlated. Full forces are retained for the UA force covariance matrix because the correlations of the covalently bonded UAs are strong and accounted for in the covariance matrix.

#### Topographical entropy

The topographical entropy for each coordinate depends on the probability of each energy well. At the M level it comprises positional entropy for translation and orientational entropy for rotation. The positional entropy arises for a dilute solute distributed among identical solvent molecules. For a molecule it is calculated by discretizing the volume *V*° available to the molecule at its concentration by the volume of a solvent molecule *V*_solvent_7
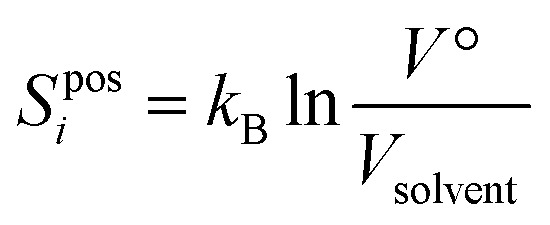
*V*_solvent_ is calculated as the volume of the simulation box of pure solvent divided by the number of solvent molecules. The logarithm is thus taken of the number of solute positions, each position has the same probability, and the larger the solvent molecule, the smaller *S*^pos^_*i*_. The choice of *V*° is not important and cancels in the calculation of log *P*, such that the change in positional entropy for the transfer is Δ*S*^pos^_*i*_ = *k*_B_ln(*V*_wat_/*V*_tol_) for all solutes. *S*^pos^_*i*_ of a pure liquid is zero because *V*° = *V*_solvent_, and is negligible for solvent in a dilute solution for a similar reason.

To calculate the orientational entropy of molecule i, its rotational volume is discretized by the number of neighbouring molecules in the first solvation shell, *N*_c_,^29,30^ weighted by the probability, *p*(*N*_c_), of each *N*_c_8
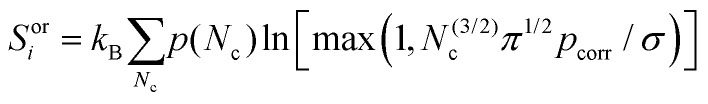
where *σ* is the symmetry number of the molecule, max ensures there is at least one orientation, and *p*_corr_ is the probability of the neighboring molecule having a compatible orientation. The respective values of *σ* for the solutes, water and toluene are 1, 2 and 2. *p*_corr_ is taken as 1 for toluene because of its weak non-bonded interactions, and 0.25 for water because there is a 0.5 probability that each of the two hydrogen bonds per water molecule are correctly aligned. Eqn (8) assumes that all orientations have equal probability. *N*_c_ is calculated using the RAD method.^42^

At the UA level, the only topographical entropy considered here is the conformational entropy, which is tantamount to UA positional entropy. For each dihedral, comprising the set of four adjacent UAs, conformers are adaptively defined from the maxima in their dihedral-angle probability distributions, however many there are.^30^ Probabilities *p*_*ik*_ are calculated for the occurrence of all unique combinations of conformers *k* over all dihedrals of molecule *i* in the MD simulation, and its conformational entropy is calculated using9
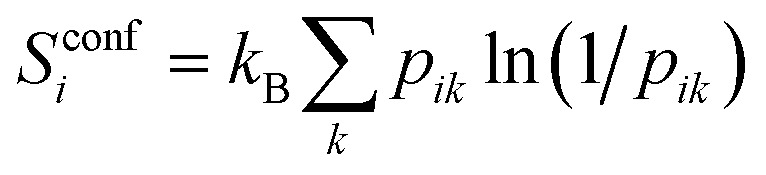
Only dihedrals that have more than one conformer contribute to *S*^conf^_*i*_. This combinatoric approach is tractable for the 3 to 6 flexible dihedral angles found in the solutes here. Water and toluene have no conformational entropy. Rotational topographical entropy of UAs is assumed to be negligible due to rigidity, symmetry, or strong correlation with the solvent, although this assumption may not hold so well for polar OH groups in toluene.

### 2.3 Model setup

The SMILES strings of the 16 drug molecules were taken from the SAMPL9 challenge website.^45^ Hydrogen atoms were added using Dimorphite-DL^46^ with the molecules having a neutral charge as instructed, as drawn in Fig. 1. Each molecular structure was optimized using autodE^47^ in the Orca v-5.0^48^ software with the PBE0/6-311G* level of density functional theory (DFT).^49^ The lowest energy conformer was selected and converted to pdb format using RDKit.^50^ The topology and coordinate files for each system were prepared using LEaP in AmberTools22.^51^ The toluene and drug molecules were modelled using the second generation general Amber force field (GAFF2)^52^ with AM1-BCC charges, and TIP3P^53^ was used for water. GAFF2 parameters were generated using the Antechamber^54^ and Parmchk2 modules of AmberTools20. Four kinds of MD simulation were set up and run: (i) 1500 water molecules, (ii) 500 toluene molecules, (iii) a single drug molecule solvated in 1500 water molecules, and (iv) a single drug molecule solvated in 500 toluene molecules. This gives 34 different simulations in total. Solvent was added using Packmol^55^ in a periodic cubic box with side ∼38 Å.

### 2.4 Molecular dynamics simulations

All MD simulations were carried out using the Particle Mesh Ewald Molecular Dynamics (PMEMD) module of the AMBER 22 software. The systems were minimized using 2000 steps of steepest decent minimization. They were heated to 298.15 K for 400 ps in the *NVT* ensemble (constant number, volume, temperature) using a Langevin thermostat^56^ with a collision frequency of 5 ps^–1^, followed by 1 ns of *NPT* simulation (constant number, volume, pressure) using the Berendsen barostat.^57^ Three production runs of 200 ns *NPT* were carried out to provide an estimate of the standard error of the mean, which contrasts with triplicates of shorter 20 ns *NPT* simulations in our original SAMPL9 submission. Altogether this gives a total of 102 simulations. All simulations used a 2 fs time step, SHAKE to constrain hydrogen atoms, and a 10 Å non-bonded cut-off. Output forces and coordinates were stored every 100 ps, giving 2000 frames. The internal entropies of the solutes were evaluated with the CodeEntropy software.^58^ The entropy of the solvent and orientational entropy of the solutes were evaluated with the same in-house C++ code used previously for liquids.^59^ This code only allowed for averaging solvent entropy over all solvent molecules. This two-part analysis was necessary because CodeEntropy does not currently include the orientational entropy for solutes or have capability for solvents other than water.

### 2.5 Error analysis

Standard errors of the mean (SEM) are calculated from the standard deviation σ using the *n* = 3 MD simulations as10
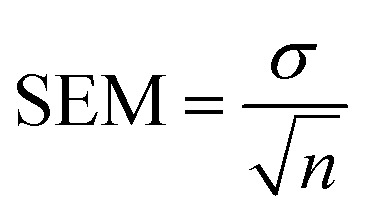
The mean unsigned error (MUE) for all drugs with respect to the experimental values is calculated using11
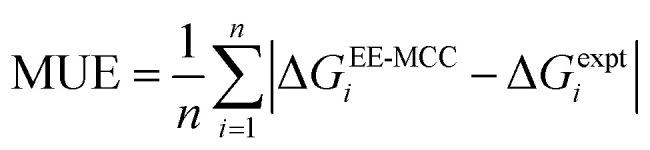
where *n* = 16 is the number of drugs.

## 3. Results and discussion

### 3.1 log *P* prediction *versus* experiment

**Fig. 2:**
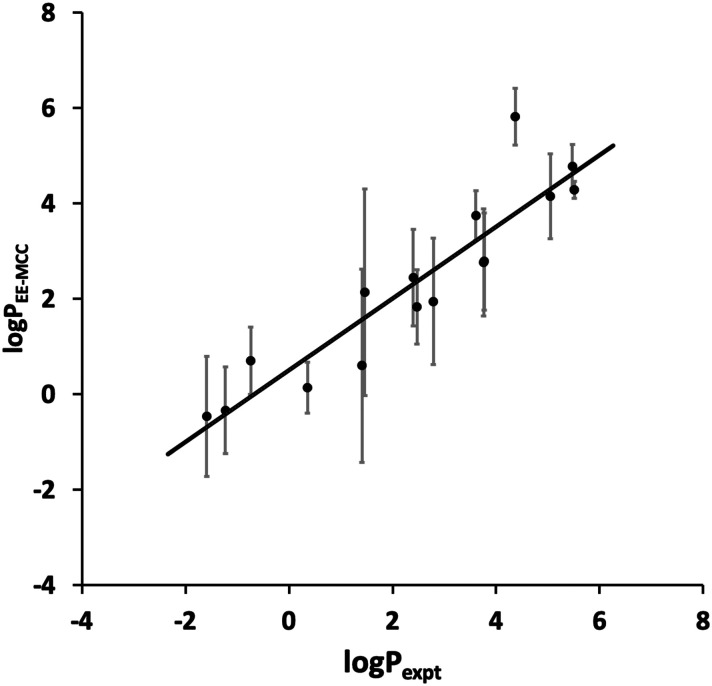
EE-MCC toluene–water log *P versus* experiment with solid line of best fit and with error bars equal to the standard error of the mean (SEM).

The toluene–water log *P* values calculated using EE-MCC for the 16 drug molecules are plotted *versus* experiment in Fig. 2. The MUE for log *P* is 0.82, the SEM is 0.97, and the slope of the line of best fit is 0.75.

Compared to the log *P* results contributed by other methods in the SAMPL9 Challenge,^45^ our results are extremely promising and would lie at the top of the list for SEM and second MUE. Other methods contributed include MM/PBSA (molecular mechanics/Poisson Boltzmann surface area), empirical methods, various electronic structure methods,^60^ non-equilibrium fast growth,^61^ free energy perturbation and RISM. Our results here use the same method as in our origional submission but with longer 200 ns simulations compared to 20 ns, which had given a larger SEM of 2.1 and MUE of 1.8.

Most drugs in Fig. 2 have SEMs close to the line of best fit, the worst outlier being amitryptiline (3) whose predicted log *P* of 5.8 makes it too hydrophobic. Nonetheless, the slope being smaller than 1 implies that MCC does not capture the full spread of log *P*. This could be because the solvent entropy was only included for the first shell and averaged over all solvent rather than for all the solvent molecules, a step taken because of the poor convergence over so many molecules and limitations in the software used. It is known that the second solvation shell and beyound can make a small but non-negligible contribution to entropy.^36,62–64^

The values of Δ*G*^transfer^_tol–wat_, Δ*H*^transfer^_tol–wat_, and *T*Δ*S*^transfer^_tol–wat_ calculated by EE-MCC are listed in Table 1 with their SEMs, together with the corresponding log *P* values by EE-MCC and experiment. Enthalpy generally contributes more to log *P* than entropy. This is reflected in the correlation coefficient, which is 0.99 for Δ*H*^transfer^_tol–wat_*versus* Δ*G*^transfer^_tol–wat_ but only 0.35 for *T*Δ*S*^transfer^_tol–wat_*versus* Δ*G*^transfer^_tol–wat_. Both plots for these correlations are illustrated in Fig. S3 (ESI†). The greater contribution of energy to log *P* may reflect the comparatively strong performance in SAMPL9 of the MM/PBSA method,^65^ which also has an energy term. This indicates that solute partitioning between water and toluene is governed primarily by enthalpy and that the overall entropy change is small. What changes there are in the entropy components tend to cancel out, especially between vibrational and topographical components for both solute and solvent. Nonetheless, the solvent entropy calculation could still be improved, as discussed earlier.

**Table 1:** EE-MCC energies (kcal mol^−1^) and log *P versus* experimental log *P* for the 16 drugs

Drug	Δ*G*^transfer^_tol–wat_	Δ*H*^transfer^_tol–wat_	*T*Δ*S*^transfer^_tol–wat_	log *P*^EE–MCC^_tol–wat_	log *P*^Expt^_tol–wat_
1	−3.8 ± 1.5	−4.2 ± 1.5	−0.4 ± 0.1	2.8 ± 1.1	3.8
2	−3.3 ± 1.4	−3.5 ± 1.5	−0.2 ± 0.1	2.4 ± 1.0	2.4
3	−5.8 ± 0.2	−5.9 ± 0.3	0.0 ± 0.1	4.3 ± 0.2	5.5
4	−6.5 ± 0.6	−6.8 ± 0.5	−0.3 ± 0.2	4.8 ± 0.5	5.5
5	−5.1 ± 0.7	−5.4 ± 0.7	−0.3 ± 0.1	3.7 ± 0.5	3.6
6	0.5 ± 1.2	1.3 ± 1.3	0.9 ± 0.1	−0.3 ± 0.9	−1.2
7	−7.9 ± 0.8	−7.4 ± 0.9	0.6 ± 0.3	5.8 ± 0.6	4.4
8	−2.6 ± 1.8	−3.3 ± 1.4	−0.7 ± 0.8	1.9 ± 1.3	2.8
9	−5.7 ± 1.2	−5.4 ± 1.3	0.3 ± 0.3	4.1 ± 0.9	5.1
10	−2.5 ± 1.1	−2.1 ± 0.9	0.4 ± 0.2	1.8 ± 0.8	2.5
11	−2.9 ± 2.9	−3.0 ± 2.9	−0.1 ± 0.1	2.1 ± 2.2	1.5
12	0.6 ± 1.7	1.1 ± 1.7	0.5 ± 0.1	−0.5 ± 1.3	−1.6
13	−0.2 ± 0.7	−0.2 ± 0.9	0.0 ± 0.2	0.1 ± 0.5	0.4
14	−0.8 ± 2.8	−0.4 ± 2.8	0.4 ± 0.2	0.6 ± 2.0	1.4
15	−1.0 ± 1.0	0.2 ± 0.9	1.2 ± 0.1	0.7 ± 0.7	−0.7
16	−3.8 ± 1.4	−3.5 ± 0.9	0.3 ± 0.1	2.8 ± 1.0	3.8

Most Δ*H*^transfer^_tol–wat_ values are negative, indicating that these molecules form stronger interactions with the non-polar solvent, being more non-polar themselves and more disruptive to the hydrogen-bond network of water than they are to toluene-toluene interactions. The exceptions to this trend are epinephrine (6), paracetamol (12) and sulfamethazine (15), which have positive Δ*H*^transfer^_tol–wat_ values, presumably because these more polar molecules lose more polar interactions with water. *T*Δ*S*^transfer^_tol–wat_ values are smaller but roughly correlated with Δ*H*^transfer^_tol–wat_ (Pearson correlation coefficient 0.53), being positive for more polar drugs and negative for less polar drugs. The average SEM over all drugs for Δ*G*^transfer^_tol–wat_ is 1.3 kcal mol^−1^, for Δ*H*^transfer^_tol–wat_ is 1.3 kcal mol^−1^ and for *T*Δ*S*^transfer^_tol–wat_ is 0.2 kcal mol^−1^. The major contribution to the error in log *P* comes from enthalpy rather than entropy. While the energies of the systems appear well converged over 200 ns of MD (Fig. S1 and S2, ESI†), these errors emphasise that EE methods require a high level of convergence to be quantitatively accurate, requiring longer simulations and more frequent data collection to bring these errors down.

### 3.2 MCC entropy components

**Fig. 3:**
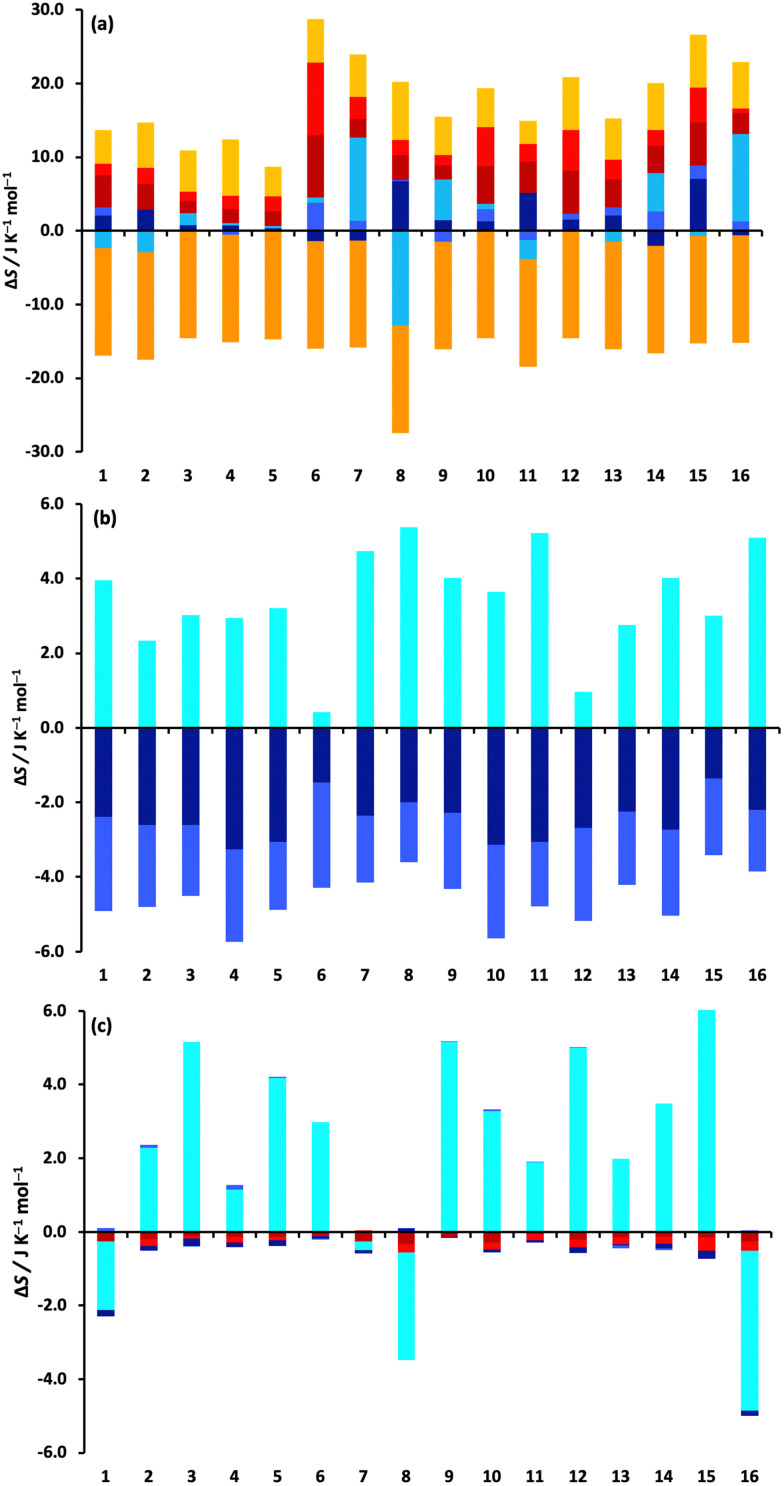
Changes in entropy components for the 16 drugs according to molecule, level and coordinate. (a) Drug, (b) water solvent, and (c) toluene solvent. The colouring of the components is according to the two levels: monomer transvibrational (dark red), rovibrational (red), positional (orange) and orientational (light orange), and united-atom transvibrational (dark blue), rovibrational (blue), positional or conformational (aqua), and orientational (cyan).

The changes in the MCC entropy components for each drug are illustrated in Fig. 3.

They give a better understanding of how the entropy changes are distributed in the system. Fig. 3a shows the components of the drug molecule. Note that the colouring scheme in Fig. 3 according to level is different to that in previous work,^59^ which coloured according to molecule level and smaller levels. Most solute entropy components are seen to increase in toluene, especially the vibrational components, which is in line with the weaker molecular interactions in toluene that would permit greater flexibility. This increase is greatest for the more polar solutes which are more confined in water than in toluene, explaining the positive *T*Δ*S*^transfer^_tol–wat_ values observed earlier in Table 1. The main exception to these component increase is Δ*S*^pos^_*i*_ which is a constant negative value for all drugs because there are fewer solute positions in toluene owing to the larger size of the toluene molecule (175.6 Å^3^*versus* 30.4 Å^3^ for water). Δ*S*^or^_*i*_ of the drugs is positive and moderately sized, indicating more toluene molecules are included in the drug solvation shell. Δ*S*^conf^_*i*_ is small for most drugs but does have large increases for fluphenazine dihydrochloride (7) and trazadone hydrochloride (16) and a large decrease for glyburide (8), suggesting the former two are more compact in water and the latter, being more polar, is more compact in toluene. Fig. 3b and c indicate the respective total contributions from water and toluene. The water contribution for solute removal is fairly uniform, comprising a decrease in vibrational entropy and stronger interactions and an increase in orientational entropy, with the exception of the two small polar drugs epinephrine (6) and paracetamol (12), although changes in orientational entropy are smaller in size compared to previous work,^33–36^ likely because of averaging first-shell values over the whole solution, as discussed earlier. The toluene contribution for solute transfer has a negligible loss of vibrational entropy and a moderate gain in Δ*S*^or^_*i*_ except for albendazole (1), glyburide (8) and trazadone hydrochloride (16).

Absolute values of the entropy components are listed in Tables S1 and S2 (ESI†) and illustrated in Fig. S4 (ESI†) for drugs in water and toluene, except for *S*^pos^_*i*_ which is concentration-dependent, a dependence that cancels for log *P* because the concentrations are the same in each liquid. Solute UA entropy scales with drug size as expected while M entropy is similar for all drugs. They also show how vibrational entropy is generally smaller for more polar drugs because of their stronger interactions.

## Conclusions

The EE-MCC method has been applied to calculate the toluene–water partition coefficients of 16 drug molecules in the SAMPL9 log *P* Challenge. For this dataset MCC is able to predict log *P* values with an average SEM error of 0.82 and of 1.3 kcal mol^−1^ for the corresponding Δ*G*^transfer^_tol–wat_. This is comparable to the best methods entered in SAMPL9 once it makes use of simulations of sufficient length, namely 200 ns *versus* 20 ns that we had used in our original submission. The main causes of error are likely the force-field, the neutral protonation states, large statistical fluctuations over many molecules, and the approximations used in MCC such as the harmonic approximation, or using solvation-shell entropy that is averaged over all the solvent. Addressing these causes will be helped in future by more frequent data saving, longer simulations, the inclusion of conformational entropy for OH and other asymmetric UAs, noise reduction, and software that enables better selection of solvent perturbed by the solute. Given that the EE-MCC method requires the difference of large numbers with non-negligible statistical errors and that there are inevitable approximations in the entropy theory, it may not always be as accurate as alchemical or knowledge-based methods. However, its ability to explain the value of the entropy from a single MD simulation of in principle any molecular system in terms of all its atomic degrees of freedom can greatly enhance the utility of simulation methods beyond what is experimentally measureable to explain molecular behaviour and guide system design.

## Author contributions

The manuscript was written by both authors, and they have given their approval to its final version.

## Conflicts of interest

There are no conflicts to declare.

## Supplementary Material

CP-025-D3CP03076H-s001
