# Supplementary material for: Energy-entropy multiscale cell correlation method to predict toluene–water log P in the SAMPL9 challenge
Source: Phys Chem Chem Phys. 2023 Sep 25;25(40):27524–31. doi: 10.1039/d3cp03076h (PMC11411597; doi:10.1039/d3cp03076h)
Supplement: CP-025-D3CP03076H-s001 [file CP-025-D3CP03076H-s001.pdf]

Electronic Supplementary Material (ESI) for Physical Chemistry Chemical Physics.
This journal is © the Owner Societies 2023

Supplementary Information

Energy-Entropy Method with Multiscale Cell Correlation to Predict Toluene-Water logP in the SAMPL9 Challenge.

Hafiz Saqib Ali[*a] and Richard H. Henchman[*b]

[a] Chemistry Research Laboratory, Department of Chemistry and the INEOS Oxford Institute for Antimicrobial Research, University of Oxford, 12 Mansfield Road, Oxford, OX1 3TA, UK
[b] Sydney Medical School, Faculty of Medicine and Health, University of Sydney, Sydney, 2006 NSW, Australia

Table S1. Entropy Components (J K^{-1} mol^{-1}) of Pure Water and of the Solute and Averaged Over All Solvent for Each Aqueous Solution

Drug	Solute						Solvent		
	$S_{\mathrm{M}}^{\mathrm{transvib}}$	$S_{\mathrm{M}}^{\mathrm{rovib}}$	$S_{\mathrm{M}}^{\mathrm{or}}$	$S_{\mathrm{UA}}^{\mathrm{transvib}}$	$S_{\mathrm{UA}}^{\mathrm{rovib}}$	$S_{\mathrm{UA}}^{\mathrm{conf}}$	$S_{\mathrm{wat,UA}}^{\mathrm{transvib}}$	$S_{\mathrm{wat,UA}}^{\mathrm{rovib}}$	$S_{\mathrm{wat,UA}}^{\mathrm{or}}$
-	-	-	-	-	-	-	47.1	21.0	10.0
1	86.9	82.6	21.6	498.8	174.1	26.8	47.5	21.4	9.3
2	87.1	83.4	19.5	519.7	269.2	44.0	47.5	21.4	9.5
3	89.5	85.5	22.5	571.9	258.2	28.8	47.6	21.4	9.4
4	89.8	84.9	19.0	656.7	216.1	35.7	47.7	21.5	9.4
5	89.6	85.8	23.5	535.9	213.0	35.8	47.6	21.3	9.4
6	81.9	74.8	21.2	341.3	163.4	17.1	47.3	21.5	9.9
7	90.5	87.4	19.6	909.4	283.1	59.1	47.5	21.3	9.1
8	88.9	87.4	15.9	1048.9	319.1	80.0	47.4	21.3	9.0
9	89.1	85.1	23.0	571.4	265.4	38.0	47.5	21.4	9.2
10	86.0	80.9	20.5	515.8	169.2	32.9	47.6	21.4	9.3
11	87.3	82.5	21.3	446.5	143.0	17.8	47.6	21.3	9.1
12	82.9	75.4	21.2	268.8	114.1	9.2	47.5	21.4	9.8
13	86.1	82.4	19.6	497.5	236.7	28.8	47.5	21.3	9.5
14	88.4	86.0	19.2	696.0	271.4	20.3	47.6	21.4	9.2
15	85.2	79.9	21.3	542.1	179.0	25.6	47.3	21.4	9.4
16	89.4	87.8	18.1	763.8	238.4	45.6	47.4	21.3	9.1

Table S2. Entropy Components (J K^{-1} mol^{-1}) of Pure Toluene and of the Solute and Averaged Over All Solvent for Each Toluene Solution

Drug	Solute						Solvent				
	$S_{\mathrm{M}}^{\mathrm{transvib}}$	$S_{\mathrm{M}}^{\mathrm{rovib}}$	$S_{\mathrm{M}}^{\mathrm{or}}$	$S_{\mathrm{UA}}^{\mathrm{transvib}}$	$S_{\mathrm{UA}}^{\mathrm{rovib}}$	$S_{\mathrm{UA}}^{\mathrm{conf}}$	$S_{\mathrm{tol,M}}^{\mathrm{transvib}}$	$S_{\mathrm{tol,M}}^{\mathrm{rovib}}$	$S_{\mathrm{tol,M}}^{\mathrm{or}}$	$S_{\mathrm{tol,UA}}^{\mathrm{transvib}}$	$S_{\mathrm{tol,UA}}^{\mathrm{rovib}}$
-	-	-	-	-	-	-	72.4	59.9	23.8	36.6	28.0
1	91.3	84.2	26.2	500.9	175.1	24.5	72.4	59.9	23.6	36.6	28.1
2	90.5	85.6	25.7	522.6	269.2	41.1	72.4	59.9	24.1	36.6	28.0
3	91.1	86.7	28.1	572.6	258.3	30.4	72.4	59.9	24.5	36.6	28.0
4	91.7	86.7	26.7	657.4	215.6	36.0	72.4	59.9	24.0	36.6	28.1
5	91.6	87.9	27.6	536.3	212.8	36.1	72.4	59.9	24.4	36.6	28.0
6	90.4	84.8	27.2	339.9	167.2	17.8	72.4	59.9	24.2	36.6	28.0
7	93.1	90.4	25.4	908.1	284.4	70.4	72.4	59.9	23.8	36.6	28.0
8	92.3	89.4	23.8	1055.6	319.3	67.1	72.4	59.9	23.4	36.6	28.0
9	91.0	86.6	28.2	572.8	263.9	43.6	72.4	59.9	24.5	36.6	28.0
10	91.2	86.2	25.7	517.1	170.8	33.7	72.4	59.9	24.3	36.6	28.0
11	91.6	84.9	24.4	451.6	141.7	15.2	72.4	59.9	24.1	36.6	28.0
12	88.8	81.0	28.3	270.3	114.8	9.3	72.4	59.9	24.5	36.6	28.0
13	89.9	85.1	25.2	499.6	237.7	27.3	72.4	59.9	24.1	36.6	28.0
14	92.1	88.1	25.6	694.0	274.1	25.5	72.4	59.9	24.3	36.6	28.0
15	91.1	84.6	28.5	549.2	180.8	24.9	72.4	59.9	24.6	36.6	28.0
16	92.3	88.5	24.4	763.2	239.7	57.4	72.4	59.9	23.3	36.6	28.0

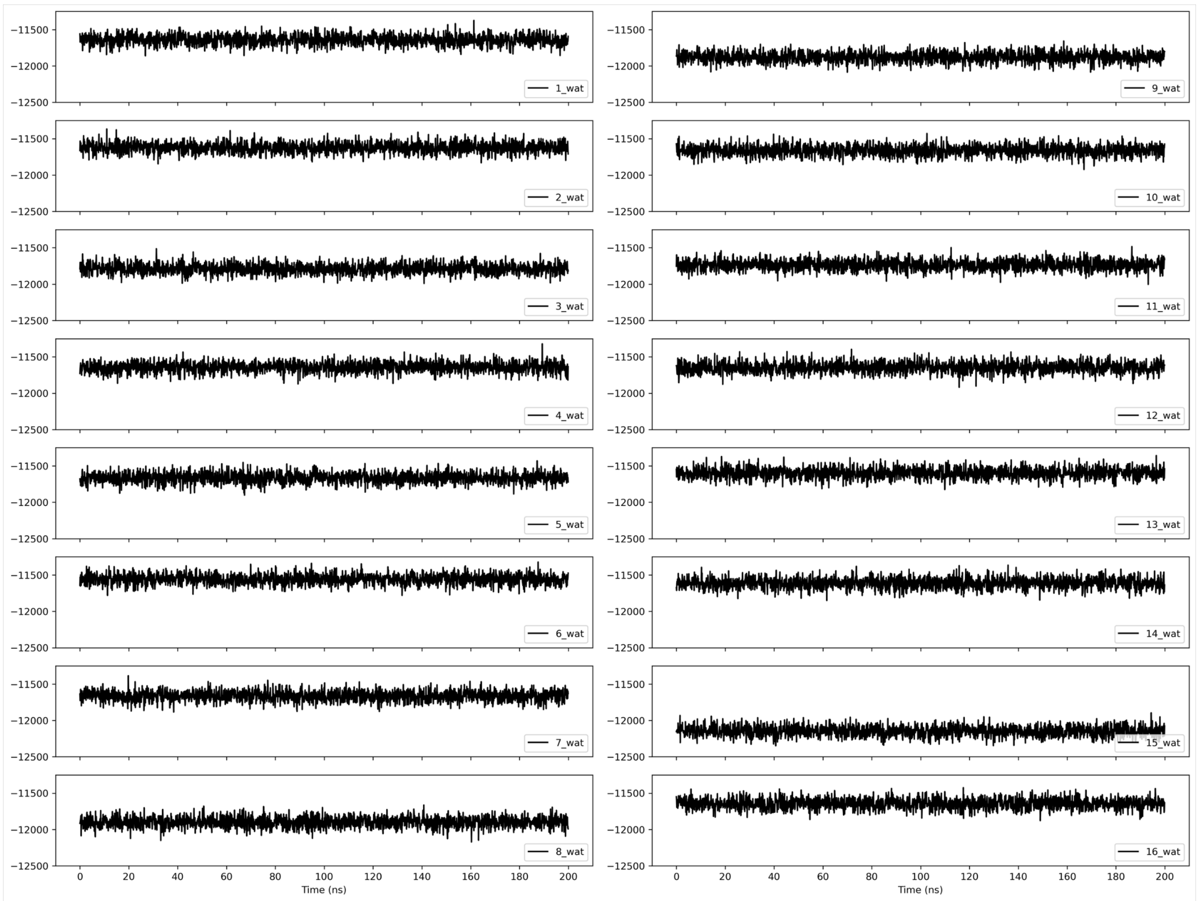

Figure S1. Energy versus simulation time for the 16 drug molecules in water.

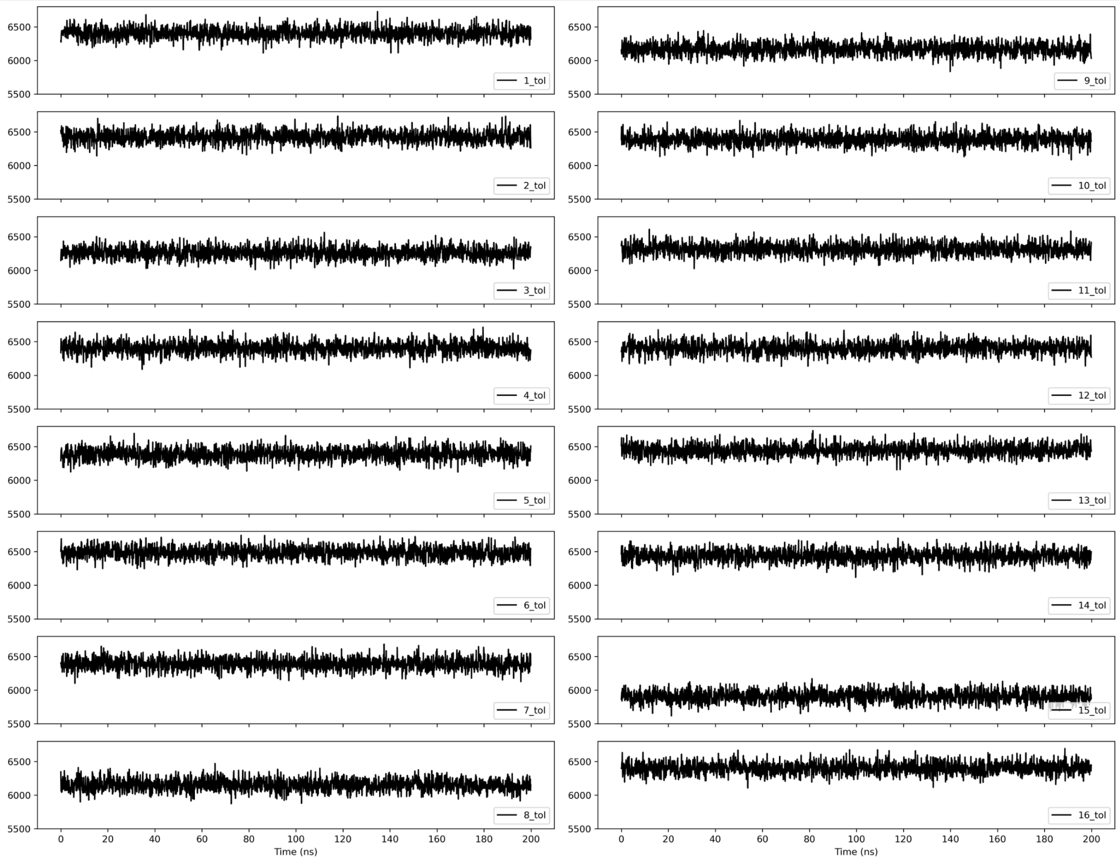

Figure S2. Energy versus simulation time for the 16 drug molecules in toluene.

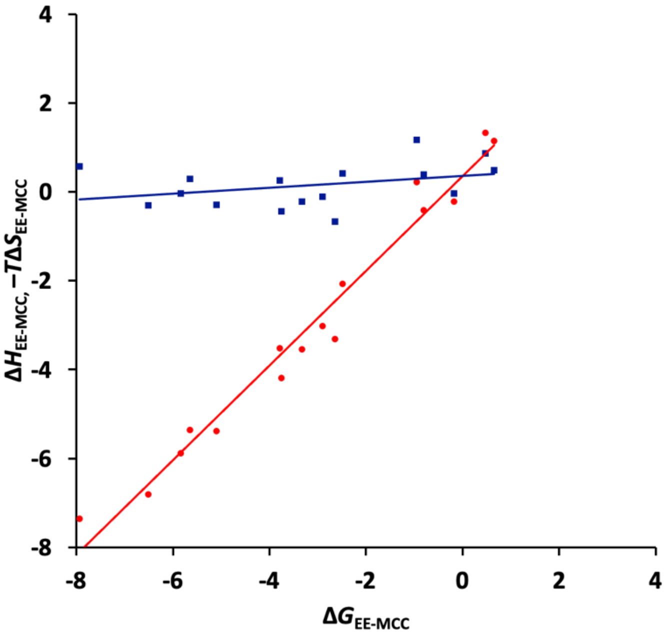

Fig. S3. $\Delta G_{EE\text{-}MCC}$ versus $\Delta H_{EE\text{-}MCC}$ (red) and $\Delta G_{EE\text{-}MCC}$ versus $-T\Delta S_{EE\text{-}MCC}$ (blue) for the transfer of the 16 drug molecules from water to toluene, including the corresponding lines of best fit.

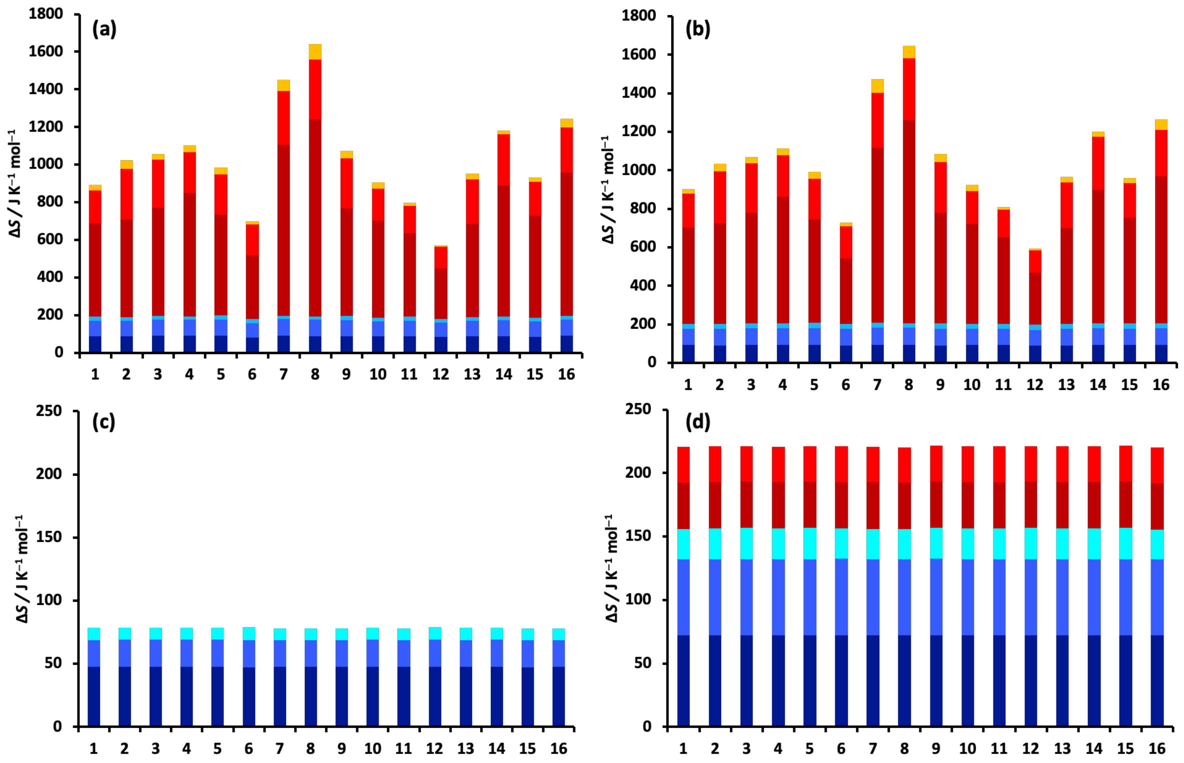

Fig. S4. MCC entropy components for the (a) drugs solvated in water, (b) drugs solvated in toluene, (c) pure water and (d) pure toluene. The colour scheme is as in **Fig. 3**.
